# Cardiovascular magnetic resonance-derived left ventricular mechanics—strain, cardiac power and end-systolic elastance under various inotropic states in swine

**DOI:** 10.1186/s12968-020-00679-z

**Published:** 2020-11-30

**Authors:** A. Faragli, R. Tanacli, C. Kolp, D. Abawi, T. Lapinskas, C. Stehning, B. Schnackenburg, F. P. Lo Muzio, L. Fassina, B. Pieske, E. Nagel, H. Post, S. Kelle, A. Alogna

**Affiliations:** 1 Department of Internal Medicine and Cardiology, Charité-Universitätsmedizin Berlin, Campus Virchow-Klinikum, Augustenburger Platz 1, 13353 Berlin, Germany; 2 Berlin Institute of Health (BIH), Berlin, Germany; 3 DZHK (German Centre for Cardiovascular Research), partner site, Berlin, Germany; 4 Department of Internal Medicine/Cardiology, Deutsches Herzzentrum Berlin, Augustenburger Platz 1, 13353 Berlin, Germany; 5 Department of Cardiology, Medical Academy, Lithuanian University of Health Sciences, Eiveniu Street 2, 50161 Kaunas, Lithuania; 6 Clinical Science, Philips Healthcare, Röntgenstr. 24, 22335 Hamburg, Germany; 7 Department of Surgery, Dentistry, Paediatrics and Gynaecology, University of Verona, Via S. Francesco 22, 37129 Verona, Italy; 8 Department of Medicine and Surgery, University of Parma, Via Gramsci 14, 43126 Parma, Italy; 9 Department of Electrical, Computer and Biomedical Engineering (DIII), Centre for Health Technologies (CHT), University of Pavia, Via Ferrata 5, 27100 Pavia, Italy; 10 Institute of Experimental and Translational Cardiac Imaging, DZHK Centre for Cardiovascular Imaging, Goethe University Hospital Frankfurt, Frankfurt am Main, Germany; 11 Department of Cardiology, Contilia Heart and Vessel Centre, St. Marien-Hospital Mülheim, 45468 Mülheim, Germany

**Keywords:** Cardiovascular magnetic resonance, Feature tracking, Left ventricular strain, Contractile function, Porcine model, Translational studies, Hemodynamics

## Abstract

**Background:**

Cardiovascular magnetic resonance (CMR) strain imaging is an established technique to quantify myocardial deformation. However, to what extent left ventricular (LV) systolic strain, and therefore LV mechanics, reflects classical hemodynamic parameters under various inotropic states is still not completely clear. Therefore, the aim of this study was to investigate the correlation of LV global strain parameters measured via CMR feature tracking (CMR-FT, based on conventional cine balanced steady state free precession (bSSFP) images) with hemodynamic parameters such as cardiac index (CI), cardiac power output (CPO) and end-systolic elastance (Ees) under various inotropic states.

**Methods:**

Ten anaesthetized, healthy Landrace swine were acutely instrumented closed-chest and transported to the CMR facility for measurements. After baseline measurements, two steps were performed: (1) dobutamine-stress (Dobutamine) and (2) verapamil-induced cardiovascular depression (Verapamil). During each protocol, CMR images were acquired in the short axisand apical 2Ch, 3Ch and 4Ch views. MEDIS software was utilized to analyze global longitudinal (GLS), global circumferential (GCS), and global radial strain (GRS).

**Results:**

Dobutamine significantly increased heart rate, CI, CPO and Ees, while Verapamil decreased them. Absolute values of GLS, GCS and GRS accordingly increased during Dobutamine infusion, while GLS and GCS decreased during Verapamil. Linear regression analysis showed a moderate correlation between GLS, GCS and LV hemodynamic parameters, while GRS correlated poorly. Indexing global strain parameters for indirect measures of afterload, such as mean aortic pressure or wall stress, significantly improved these correlations, with GLS indexed for wall stress reflecting LV contractility as the clinically widespread LV ejection fraction.

**Conclusion:**

GLS and GCS correlate accordingly with LV hemodynamics under various inotropic states in swine. Indexing strain parameters for indirect measures of afterload substantially improves this correlation, with GLS being as good as LV ejection fraction in reflecting LV contractility. CMR-FT-strain imaging may be a quick and promising tool to characterize LV hemodynamics in patients with varying degrees of LV dysfunction.

## Background

The routine assessment of left ventricular (LV) ejection fraction (LVEF), being a measure of global systolic function, falls short in identifying regional myocardial impairment and the mechanical contraction of the heart [1, 2]. Therefore, strain imaging has emerged in the past years to better quantify myocardial LV deformation in various patient populations [3–7]. Numerous studies have validated and shown the utility of myocardial strain in the diagnosis of several pathologies, identifying subclinical myocardial changes, and even by showing an impact on the prognosis of cardiovascular pathologies [8–15]. Recently, cardiovascular magnetic resonance (CMR) feature tracking (FT) strain analysis was shown to be accurate in the detection of myocardial dysfunction as well as useful as a predictor of major adverse cardiac events, with the advantage of utilizing conventional balanced steady-state free precession (bSSFP) cine sequences [15–19]. Impaired LV systolic function and cardiac reserve can be clinically assessed by hemodynamic parameters such as cardiac index (CI), as well as by cardiac power output (CPO) at rest and during pharmacological stress. In particular, the latest has been shown to strongly correlate with the clinical outcome of chronic heart failure patients [20, 21]. Moreover, LV CPO is able to provide an assessment of the intraventricular flow as well as of its mechanics much more than other hemodynamic parameters, since it couples not only with the cardiac work, but also with the response of the vasculature [22, 23]. The invasively measured end-systolic elastance (Ees) is instead, a relatively load-independent parameter describing the LV inotropic state [24]. Recently, Seeman and colleagues successfully investigated a novel CMR method to noninvasively quantify Ees [25]. Whether or not, and to what extent CMR-FT LV strain reflects the above-mentioned hemodynamic parameters under various inotropic states has not been investigated thus far. Therefore, the aim of this study was to validate the correlation of CMR LV strain parameters against hemodynamic parameters such as CI, CPO and the Ees mentioned above, under various inotropic states in swine.

## Methods

The experimental protocols were approved by the local bioethics committee of Berlin, Germany (G0138/17), and conform to the “European Convention for the Protection of Vertebrate Animals used for Experimental and other Scientific Purposes” (Council of Europe No 123, Strasbourg 1985).

### Experimental setup

Female Landrace swine (n = 10, 51 ± 10 kg) were fasted overnight with free access to water, and then sedated and intubated on the day of the experiment. Anaesthesia was continued with fentanyl, midazolam, ketamine and pancuronium as needed. The anesthesia regimen included low-dose isofluorane to obtain a deeper sedation and stabilize hemodynamics without impacting much on systemic vascular resistance. Animals were ventilated (Cato, Dräger Medical, Lubeck, Germany) with a FiO2 of 0.5, an I: E-ratio of 1:1.5, the positive end-expiratory pressure was set at 5 mmHg and a tidal volume of 10 ml kg^-1^. The respiratory rate was continuously adjusted to maintain an end-expiratory carbon dioxide partial pressure between 35 and 45 mmHg. Under fluoroscopic guidance, all animals were instrumented with a floating balloon catheter in the right atrium, as well as in the coronary sinus (Arrow Balloon Wedge-Pressure Catheters, Teleflex Inc, Wayne, Pennsylvania USA). In order to avoid CMR-artefacts, the balloon-tip was cut before introducing the catheters in the vessel. Respiratory gases (PM 8050 MRI, Dräger Medical), heart rate (HR), and invasively derived arterial blood pressure were continuously monitored (Precess 3160, InVivo, Gainesville, Florida, USA) via a sheath access surgically prepared in the internal carotid artery. Body temperature was monitored by a sublingual thermometer and was maintained at 38 °C during CMR imaging via air ventilation or infusion of cold saline solution.

### Experimental protocols

After acute instrumentation, the animals were transported to the CMR facility for measurements. After baseline measurements, two steps were performed: (1) Dobutamine-stress (Dobutamine) and (2) verapamil-induced cardiovascular depression (Verapamil). Dobutamine infusion was titrated aiming at a 25% HR increase compared to baseline values, while verapamil was given as single 2.5 mg bolus, aiming at a 25% decrease of CI. This protocol was established beforehand with a small pilot study (data not shown), in which the titration of dobutamine and verapamil was assessed by LV invasive conductance measurements according to previous publications by our group [26]. The cumulative dose for each experiment was achieved via careful titration of verapamil, administered as a 2.5 mg bolus in order to avoid a pronounced hypotension leading to hemodynamic instability. In the pilot experiments CI was continuously assessed online via a Swan-Ganz catheter in the pulmonary artery (Edwards Lifesciences CCO connected to Vigilance I, Edwards Lifesciences, Irvine, California, USA). In the CMR study, after the first bolus, we estimated stroke volume via short-axis cine imaging after reaching a hemodynamic steady state (around 5 min after bolus injection). In case CI was not decreased as much as 25%, we proceeded with a further bolus of verapamil. Between the different protocol steps there was a wash-out period of 30 min. The anaesthesia regimen included low dose isoflurane to obtain a deeper sedation and stabilize hemodynamics without impacting much on systemic vascular resistance. This protocol was established beforehand with a small pilot study (data not shown), in which the titration of dobutamine and verapamil was assessed by LV invasive conductance measurements according to previous publications by our group [26]. At each protocol step, CMR images were acquired in the short axis (SAx), two-chamber (2Ch), three-chamber (3Ch) and four-chamber (4Ch) views. At the end of the measurements, animals were transported back to the operating room for sacrifice. Sacrifice was performed with an intracoronary 80 mmol potassium bolus.

### Cardiovascular magnetic resonance

All CMR images were acquired in a supine position using a 3 T CMR scanner (Ingenia, Philips Healthcare, Best, The Netherlands) CMR scanner with an anterior- and a built-in posterior coil element, where up to 30 coil elements were employed, depending on the individual anatomy. All animals were scanned using identical comprehensive imaging protocol. The study protocol included initial scouts to determine cardiac imaging planes. Cine images were acquired using electrocardiogram (ECG)-gated bSSFP cine sequence in three LV long-axis (2Ch, 3Ch, 4Ch) planes. The ventricular 2Ch and 4Ch planes were used to plan stack of SAx slices covering entire LV. The following imaging parameters were used: repetition time (TR) = 2.9 ms, echo time (TE) = 1.45 ms, flip angle = 45°, measured voxel size = 1.9 × 1.9 × 8.0 mm^3^, reconstructed voxel size 1.0 × 1.0 × 8mm^3^, and 40 cardiac phases.

### Image analysis

All images were analyzed offline using a commercially available software (Medis Suite, version 3.1, Leiden, The Netherlands) in accordance with a recent consensus document for quantification of LV function using CMR [27]. A numeric code was assigned to the sequences of different measurements steps and the observers were therefore blinded to the pharmacological interventions. Given the excellent inter-observer reproducibility, we averaged values obtained by several measurements from one observer.

**Fig. 1:**
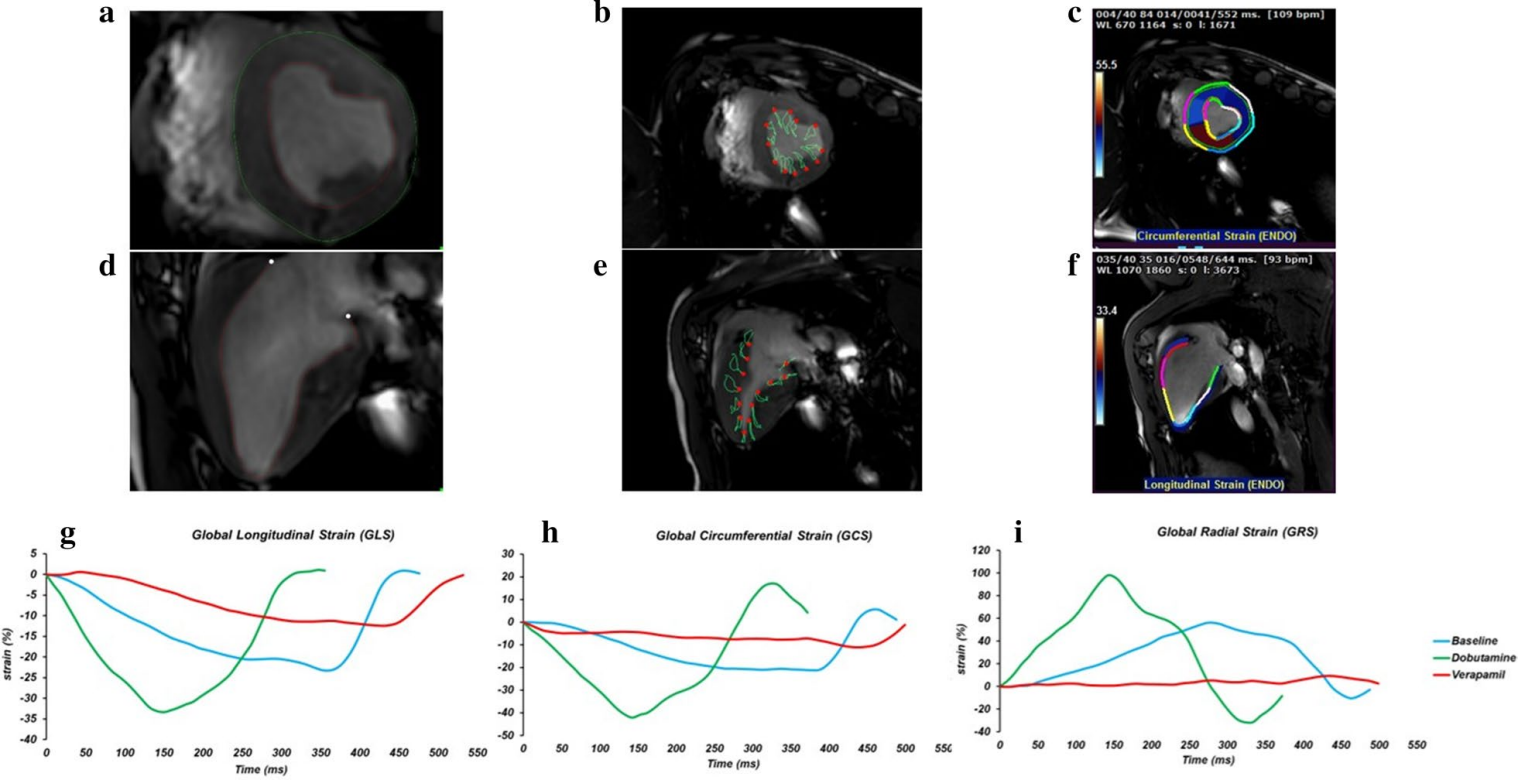
Global strain analysis via Qstrain and representative left ventricular (LV) strain curves from one experiment. **a** Short axis endocardial and epicardial contouring; **b** circumferential strain measured with Qstrain, the lower end of the scale bar is equal to zero and it is not represented in the original window from MEDIS; **c** circumferential strain measured at end-systole time-to-peak; **d** longitudinal axis endocardial contouring; **e** longitudinal strain measured with Qstrain, the lower end of the scale bar is equal to zero and it is not represented in the original window from MEDIS; **f** longitudinal strain measured at end-systole time-to-peak; **g** global longitudinal strain (GLS), **h** global circumferential strain (GCS), and **i** global radial strain (GRS) representative curves from one animal measured during Baseline, Dobutamine (Dob) and Verapamil (Ver) (mean values of all segments)

On SAx view, the outline of the endocardial border of the LV was manually traced on all slices of each phase. Volumes were computed by Simpson method of disks summation, whereby the sum of cross-sectional areas was multiplied by slice thickness (8 mm). The LVEF was calculated using the Simpson method. The LV outflow tract was included as LV blood volume. Papillary muscles and trabeculation were included as LV volume. The ascending aorta was outlined in all the images and flow calculation was performed in the corresponding velocity-encoded phase images. The average flow velocity (cm/s) was multiplied by the area of the vessel (cm^2^) to obtain the flow (ml/s) and integrated over one cardiac cycle to obtain the stroke volume (SV). Then, the cardiac output (CO) is indirectly calculated as the product of SV and HR. Finally, the CI is calculated as the CO divided by the body surface area (BSA) [28]. For the strain analysis 2Ch, 3Ch and 4Ch cine images, and respectively, 3 preselected mid-ventricle slices from the LV SAx stack were included. The endocardial and epicardial contours drawn on cine images with QMass (version 8.1, Medis Medical) were transferred to QStrain RE (version 2.0, Medis Medical) where after the application of tissue tracking algorithm, endocardial and epicardial borders were detected throughout all the cardiac cycle (Fig. 1a, d). These long-axis cine images were further used to compute myocardial global longitudinal strain (GLS), and SAx images were used to compute global circumferential strain (GCS) and global radial strain (GRS) and strain-rate (Fig. 1b, e). The global values were obtained by averaging the values of systolic peak strain according to an AHA 17 segments model, apex being excluded, as follows: GCS from averaging circumferential strain for 6 basal, 6 mid and 4 apical segmental individual values; GLS from 2Ch, 3Ch and 4Ch averaging 6 basal, 6 mid and 4 apical segments using a bull-eye view (Fig. 1c, f, g, h, i). Data on strain rate are presented in Table 3. In line with the global strain parameters, dobutamine increased peak systolic SR. Verapamil significantly decreased peak systolic SR compared to dobutamine but not to baseline values.

### Hemodynamic parameters

Systolic blood pressure (SBP), diastolic blood pressure (DBP) and mean aortic pressure (mAoP) were invasively measured throughout the entire protocol study via a sheath access surgically prepared in an internal carotid artery.

The systemic vascular resistance was calculated as follows:

$$SVR_{mmHg/L} = \frac{{Mean\,Arterial\,Pressure \left( {MAP} \right) - Right\,Atrial\,Pressure}}{{Cardiac\,Output \left( {CO} \right)}}$$

CPO, CI and Ees were calculated as follows:

$$CPO = \frac{CO \times mAoP}{{451}}$$

$$CI = \frac{CO}{{BSA}}$$

$$Ees = \frac{{LVP_{sys} }}{{V_{{LVP_{max} }} - V_{0} }}$$

where:

$$LVP_{sys} = \frac{2}{3}Systolic\,Blood\,Pressure + \frac{1}{3}Diastolic\,Blood\,Pressure$$

$$V_{{LVP_{max} }} = End - Systolic\,Volume$$

$$V_{0} = 0$$

as described in the work by Kelly et al [29].

GLS, GCS and GRS were indexed to the measured mAoP adapting the formula from the previous study by Rhea et al. [30] as follows:

$$\frac{Global\,Strain \times mAoP}{{avg\left( {mAoP} \right)}}$$

where Global Strain was the global value of either longitudinal (GLSi), circumferential (GCSi) or radial (GRSi) strain and avg(mAoP) was the average of the mAoP measured for each protocol step, namely at baseline, dobutamine and verapamil (Table 1).

**Table 1:** Systemic hemodynamics and cardiac mechanics parameters during BL, Dob and Ver steps

	Baseline	Dobutamine	Verapamil
HR (bpm)	106 ± 15	146 ± 12*	98 ± 19*^,§^
LVEF (%)	59 ± 8	77 ± 7*	39 ± 9*^,§^
CO (L/min)	6 ± 1	9 ± 2*	4 ± 1*^,§^
CI (L/min/m^2^)	2.5 ± 0.2	3.8 ± 0.5*	1.7 ± 0.7*^,§^
CPO (W)	1.2 ± 0.3	2.0 ± 0.6*	0.7 ± 0.2*^,§^
SVR (dyn s cm^−5^)	15 ± 5	11 ± 4*	19 ± 9*^,§^
mAoP (mmHg)	90 ± 12	98 ± 19	70 ± 10*^,§^
Wall stress (mmHg)	0.12 ± 0.02	0.16 ± 0.04*	0.10 ± 0.02^§^

*HR* heart rate, *LVEF* left ventricular ejection fraction, *CO* cardiac output, *CI* cardiac index, *CPO* cardiac power output, *SVR* systemic vascular resistance, *mAoP* mean aortic pressure

*p < 0.05 vs. Baseline; ^§^p < 0.05 vs. Dobutamine

Meridional wall stress was calculated via the following formula [31]:

$$LV\,wall\,stress = \frac{{\left( {0.334 \times LVP_{sys} \times LVESD} \right)}}{PWT \times [1 + PWT/LVID]}$$

where LVESD = left ventricular end-systolic diameter and PWT = posterior wall thickness as described in the paper by Reichek et al. [31]. PLT was measured by averaging three separate measurements in the basal short axis sequence.

GLS, GCS and GRS were indexed to the measured wall stress adapting the formula from the study by Reichek et al. [31] as follows:

$$\frac{Global\,Strain \times LV\,wall\,stress}{{avg\left( {LV\,wall\,stress} \right)}}$$

where Global Strain was the global value of either global longitudinal (GLSw), global circumferential (GCSw) or global radial (GRSw) strain. The average for both LV wall stress were calculated for each step, namely at baseline, dobutamine and verapamil (Table 1).

### Statistical analysis

All data are presented as mean ± SD. The association between strain data and hemodynamic data was assessed by linear regression analysis. The condition (baseline, dobutamine, verapamil) was included as a regressor into the linear regression model. To verify whether the linear regressions were significantly different (p-value < 0.05), using custom-made scripts in MATLAB (release R2020a; The MathWorks, Inc., Natick, Massachusetts, USA), we compared slopes, intercepts as well as correlation coefficients: (i) via t-test (for slopes and intercepts) and (ii) via the Fisher’s r-to-z transformation followed by z-test (for correlation coefficients), as previously described by Weaver and Wuensch [32]. Data between groups at different inotropic states were analysed by one-way ANOVA for repeated measurements. Post-hoc testing was performed by Tukey’s test. A p-value < 0.05 was considered significant. For statistical calculations, we used the software Sigmastat (Version 4.0, Systat Software, Inc., Cranes Software, Karnataka, India) and SPSS (Version 23.0, Statistical Package for the Social Sciences, International Business Machines, Inc., Armonk, New York, USA).

## Results

The dose of dobutamine needed to induce a 25% HR increase was 6.4 ± 2.5 µg/kg/min, while the dose of verapamil needed to decrease CI significantly was 5 ± 2 mg.

### Systemic hemodynamics

**Fig. 2:**
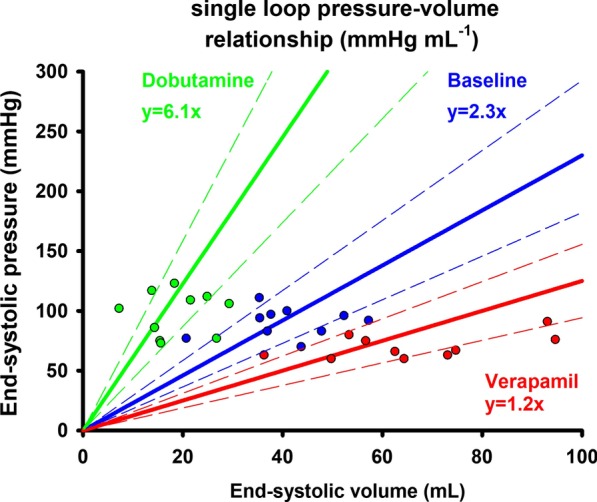
Averaged end-systolic pressure–volume relationship at baseline, during dobutamine and verapamil. Single-loop derived by the LV end-systolic pressure–volume relationship (ESPVR) is plotted under various inotropic states. The green line corresponds to the averaged ESPVR during dobutamine infusion, the blue line represents the averaged ESPVR at baseline, while the red one represented the averaged ESPVR at verapamil. A steeper increase in ESPVR during dobutamine and a relevant decrease during verapamil are observed. The equation for each ESPVR is displayed in the graph. Data points are plotted for each animal during different inotropic states. The dashed lines represent the 95% confidence intervals

Systemic hemodynamic data are summarized in Table 1. mAoP was not affected by Dobutamine, but significantly decreased during Verapamil. Dobutamine increased baseline HR, CO and LVEF, while Verapamil decreased them. Systemic vascular resistance (SVR) substantially decreased during Dobutamine, while increased during Verapamil. CPO and CI both increased during Dobutamine and decreased during Verapamil. Ees, the slope of the end-systolic pressure–volume relationship, increased during Dobutamine and decreased during Verapamil infusion (Fig. 2).

### Global strain parameters

Strain parameters are summarized in Table 2a, b and c. GLS as well as GCS increased during Dobutamine, while decreased during Verapamil. GRS was not significantly affected by Dobutamine, while decreased significantly during Verapamil.

**Table 2:** Global strain and indexed global strain values measured at different inotropic states

	Baseline	Dobutamine	Verapamil
(A)			
GLS (%)	− 23 ± 4	− 45 ± 9*	− 16 ± 3*^,§^
GCS (%)	− 31 ± 8	− 53 ± 10*	− 17 ± 5*^,§^
GRS (%)	72 ± 19	88 ± 36	30 ± 12*^,§^
(B)			
GLSi (%)	− 23 ± 4	− 45 ± 10*	− 16 ± 4*^,§^
GCSi (%)	− 30 ± 8	− 52 ± 8*	− 16 ± 5*^,§^
GRSi (%)	71 ± 19	84 ± 23	30 ± 13*^,§^
(C)			
GLSw (%)	− 23 ± 5	− 44 ± 10*	− 16 ± 3*^,§^
GCSw (%)	− 31 ± 9	− 52 ± 13*	− 17 ± 17*^,§^
GRSw (%)	71 ± 20	90 ± 54	35 ± 27*^,§^

(A) Global strain: *GLS* global longitudinal strain, *GCS* global circumferential strain, *GRS* global radial strain; (B) Global strain indexed for mean aortic pressure (mAoP): *GLSi* global longitudinal strain indexed for mAoP, *GCSi* global circumferential strain indexed for mAOP, *GRSi* global radial strain indexed for mAoP; (C) Global strain indexed for meridional wall stress: *GLSw* global longitudinal strain indexed for wall stress, *GCSw* global circumferential strain indexed for wall stress, *GRSw* global radial strain indexed for wall stress. *p < 0.05 vs. baseline; ^§^p < 0.05 vs. Dobutamine

### Systolic strain rate

Data on strain rate (SR) are presented in Table 3. In line with the global strain parameters, Dobutamine increased peak systolic SR. Verapamil significantly decreased peak systolic SR compared to Dobutamine but not to baseline values.

**Table 3:** Global peak systolic strain rates values measured at different inotropic states

	Baseline	Dobutamine	Verapamil
GLS peak systolic SR (s-1)	− 2.5 ± 0.6	− 6.4 ± 1.5*	− 2.1 ± 1.1^§^
GCS peak systolic SR (s-1)	− 3.2 ± 2.2	− 8.7 ± 2.5*	− 2.0 ± 1.3^§^
GRS peak systolic SR (s-1)	2.7 ± 1.0	5.5 ± 0.9	2.2 ± 1.5^§^

Strain Rate (SR). *p < 0.05 vs. Baseline; ^§^p < 0.05 vs. Dobutamine

### Indexing strain parameters for indirect measures of afterload

Indexing global strain parameters for either mAoP (Table 2b) or for meridional wall stress (Table 2c) did not significantly impact the above-described changes induced by Dobutamine and Verapamil.

### Correlation between global strain, LVEF and LV hemodynamic parameters

**Fig. 3:**
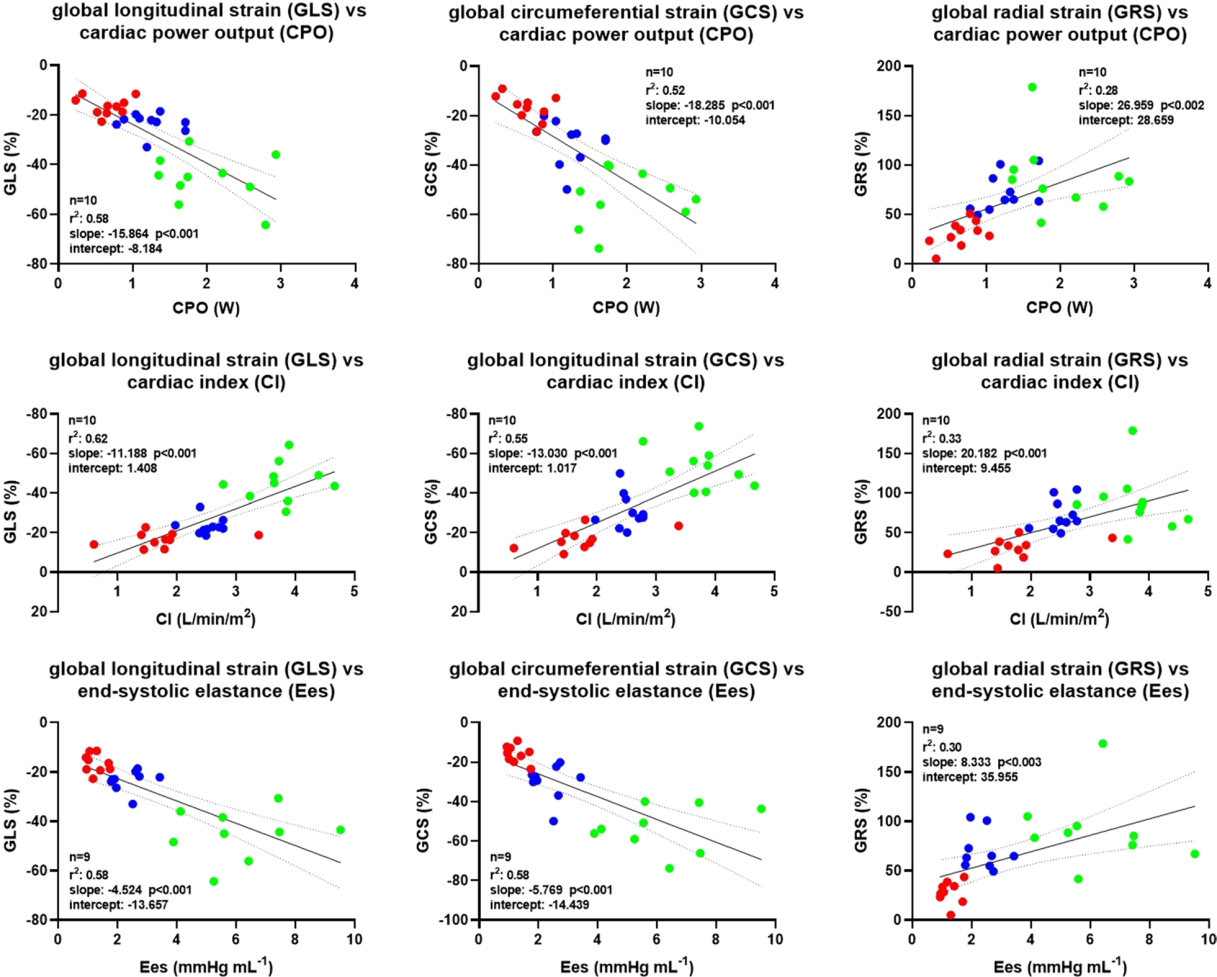
Correlation between global strain and different invasive parameters of hemodynamics. **a** Linear regression analysis showing a moderate correlation between GLS, GCS, and CI; and a poor correlation between GRS and CI. **b** Linear regression analysis showing a moderate correlation between GLS, GCS and CPO; and a poor correlation between GRS and CPO. **c** Linear regression analysis showing a moderate correlation between GLS, GCS, and Ees; and a poor correlation between GRS and Ees. Blue dots represent *BL* baseline, green dots represent *Dob* dobutamine, red dots represent *Ver* verapamil

**Fig. 4:**
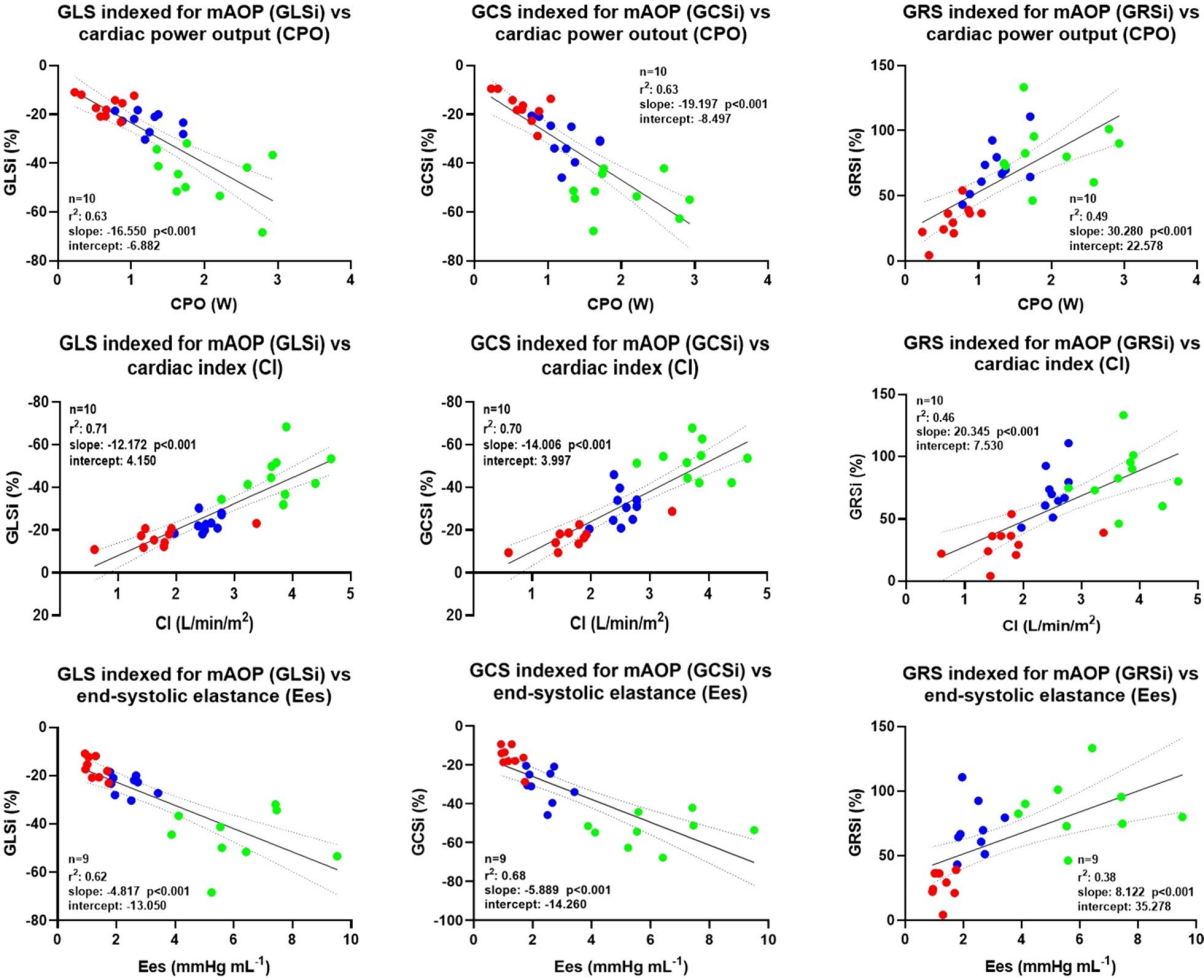
Correlation between global strain indexed for mean aortic pressure and different invasive parameters of hemodynamics. Linear correlation between GLSi, GCSi, GRSi; and CI (**a**), CPO (**b**), and Ees (**c**), after adjusting strain values for mean aortic pressure (mAoP), according to the following formula (Global Strain × mAOP/avg (mAoP). Blue dots represent *BL* baseline, green dots represent *Dob* dobutamine, red dots represent *Ver* verapamil. *GLSi* global longitudinal strain indexed for mAoP, *GCSi* global circumferential strain indexed for mAoP, *GRSi* global radial strain indexed for mAoP

**Fig. 5:**
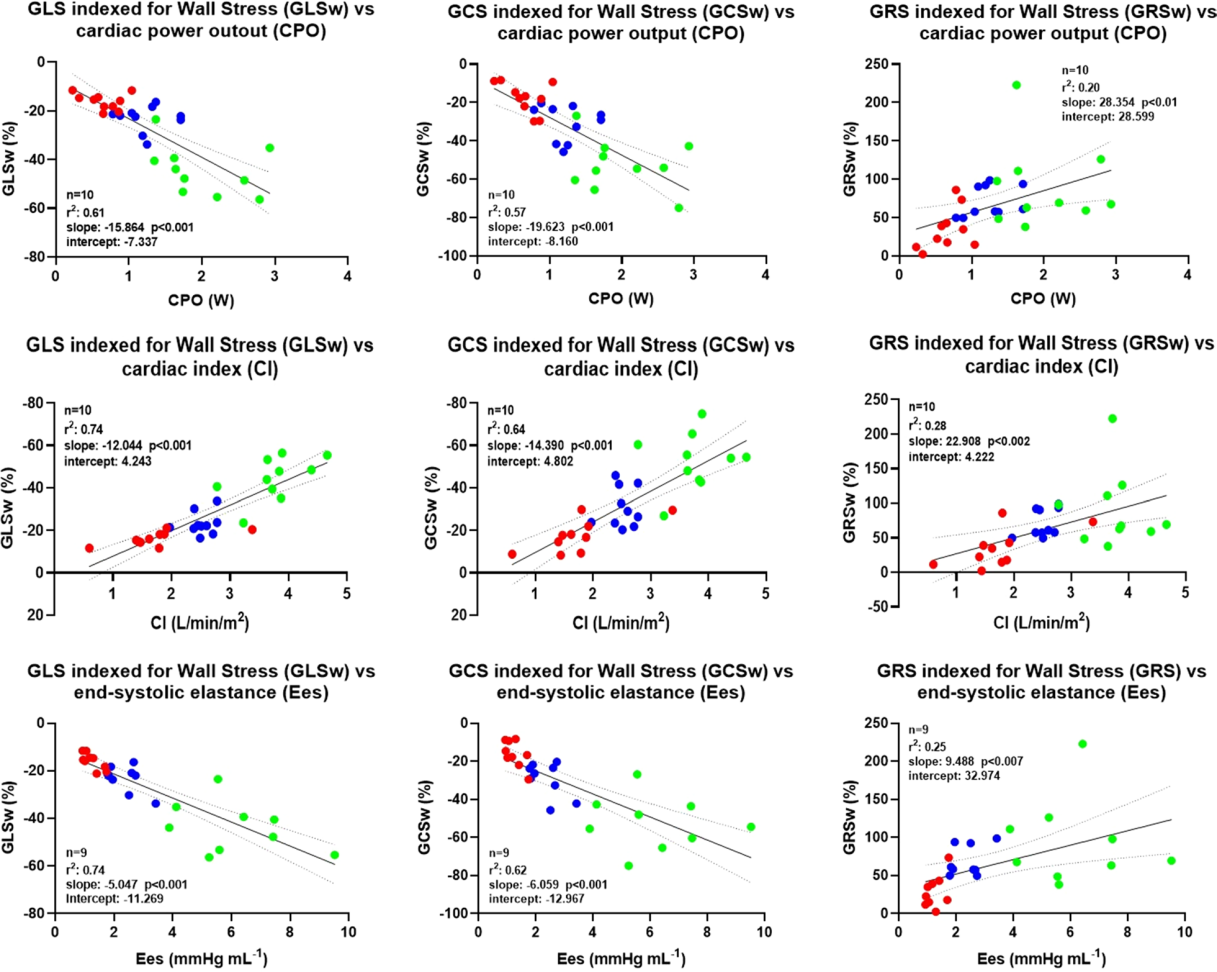
Correlation between global strain indexed for wall stress and different invasive parameters of hemodynamics. Linear correlation between GLSw, GCSw, GRSw, LVEF; and CI (**a**), CPO (**b**), and Ees (**c**), after adjusting strain values for meridional Wall Stress, according to the following formula (Global Strain × Wall Stress / avg (Wall Stress). Blue dots represent *BL* baseline, green dots represent *Dob* dobutamine, red dots represent *Ver* verapamil

Linear regression analysis showed a moderate correlation between GLS, GCS and CPO, while a poor one was observed between GRS and CPO (Fig. 3a). A similar correlation was observed between GLS, GCS and CI (Fig. 3b), with GRS worst performing. A moderate correlation was observed between GLS, GCS and Ees, while a poor one was observed between GRS and Ees (Fig. 3c). Indexing global strain parameters either for mAoP or for wall stress improved their correlations with CPO (Figs. 4a and 5a), with CI (Figs. 4b and 5b) as well as with Ees (Figs. 4c and 5c). LVEF moderately correlated with CI and CPO (r^2^ = 0.81 and r^2^ = 0.69, respectively) as GLSw with CI and CPO (r^2^ = 0.74 and r^2^ = 0.72, respectively). GLSw moderately correlated with Ees as well as LVEF with Ees (r^2^ = 0.74 versus r^2^ = 0.74). The above-mentioned correlations were both statistically significant with a p < 0.0001.

### Relative change of mechanics under various inotropic states

**Fig. 6:**
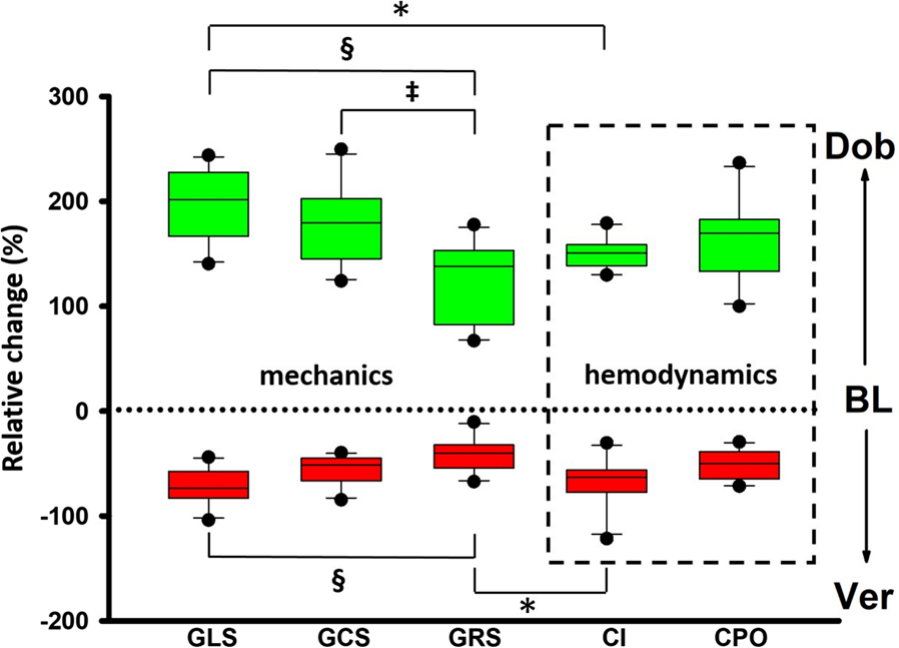
Relative change of global strain and cardiac mechanics parameters from baseline to dobutamine or verapamil. The schematic representation above shows the relative change of global strain values and invasive hemodynamic parameters (dashed box) from baseline (BL, dashed horizontal line) to dobutamine (Dob, green boxes) and from BL to verapamil (Ver, red boxes), respectively. *p < 0.05 vs CI, ^§^p < 0.05 vs GLS, ^‡^p < 0.05 vs GCS

In Fig. 6, we plotted the relative change of mechanics (global LV strain parameters) as well as hemodynamic parameters during Dobutamine and Verapamil in comparison to baseline.

Among global strain parameters, GLS showed a higher relative change than GRS during both Dobutamine and Verapamil, while the same was valid for GCS during dobutamine only. Moreover, the impact of Dobutamine was more prominently expressed by GLS than by CI, while the impact of Verapamil on GRS was negligible when in comparison to CI. The impact of Dobutamine and Verapamil on the rest of the mechanic and hemodynamic parameters was comparable.

## Discussion

CMR strain imaging is an established technique to quantify myocardial deformation. However, to what extent LV systolic strain, and therefore LV mechanics, reflects classical hemodynamic parameters under various inotropic states is still not completely clear. In the current study, we set out to investigate the correlation of LV global strain parameters measured via CMR-FT with hemodynamic parameters under various inotropic states in swine. We observed a moderate correlation of global strain parameters with LV hemodynamics. Interestingly, indexing strain parameters for indirect measures of afterload substantially improved this correlation, with GLS indexed for wall stress reflecting LV contractility as the clinically widespread LVEF.

### Correlation between global strain, LVEF and LV hemodynamic parameters

Numerous studies have reported a significant diagnostic as well as a prognostic role of LV strain in the assessment of LV mechanics in various study populations [10, 11], including patients with heart failure with preserved ejection fraction, coronary artery disease, diabetes mellitus, hypertensive heart disease, hypertrophic cardiomyopathy, and arrhythmia [1–7]. Recently, CMR-FT strain analysis was shown to be accurate in the detection of myocardial dysfunction and as well useful as a predictor of major adverse cardiac events in ischemic or non-ischemic cardiomyopathy [18], with the advantage of utilizing simple bSSFP cine sequences [15–19, 33, 34]. However, notwithstanding the multitude of published papers assessing the clinical usefulness of strain, there is a notable lack of in-vivo validation studies [35]. Whether CMR-FT LV strain represents a valid tool to assess the cardiac mechanics of the myocardium, and how this varies under different inotropic states, is still unclear. In this work, we could show a role of CMR LV strain as a surrogate of classic hemodynamic parameters such as CI, CPO, and a load-independent parameter of cardiac contractility such as Ees, which are established parameters to describe the hydraulic and mechanical role of the heart as a pump [22, 24]. Previous in vivo studies concentrated on the role of 2D and 3D speckle tracking echocardiography (STE) [35]. The main validation studies analysed the correlation of STE with the sonomicrometry technique in open-chest large animal models, showing an overall good agreement of the two techniques [35]. Similarly to our study, Weidemann et al. showed the ability of strain as well as strain rate to reflect swine LV-contractility under different inotropic states and independently of heart rate [36]. The invasively measured Ees, the slope of the end-systolic pressure–volume relationship, is a relatively load-independent parameter describing the LV inotropic state [24]. A recent study by Seeman et al. established a reliable method to assess Ees via CMR imaging [25]. In the current work, we showed a moderate to good correlation between both GLS, GCS, and Ees. The accuracy of GLS in reflecting Ees improved when indexing for wall stress. In line with our data, Yotti et al [8] showed a moderate correlation between echocardiographic assessed GCS and invasive pressure–volume catheterization data, while on the opposite, GLS correlated poorly. The authors highlighted how the GCS measurements, as opposed to the GLS, did not change in patients with aortic stenosis or hypertension. They hypothesized this could be related to a lower load-dependency [8] of the first in comparison to the latter ones, but the different methodology of strain assessment in comparison to our study is probably playing a role as well.

### Indexing strain parameters for indirect measures of afterload

In the current study, in order to minimize the load-dependency of the strain measurements, we indexed all the global strain values for indirect measures of afterload, such as the invasively measured mAoP as well as the meridional wall stress, as already described by Rhea et al. and Reichek et al [30, 31]. Correcting the strain measurements, as mentioned above, improved the ability of strain parameters to reflect LV hemodynamics. Yingchoncharoen et al. already demonstrated that blood pressure adjustment of strain is advisable in patients with large deviations of SBP from the normal-reference value [37]. Furthermore, in a study by Weiner et al. on the impact of isometric handgrip testing on LV twist mechanics, it was shown that longitudinal strain is influenced by blood pressure [38]. Finally, in line with our study, Rhea et al. showed an improved accuracy of pressure-adjusted GLS in predicting cardiac events and mortality [30].

### Relative change of mechanics under various inotropic states

In order to investigate to what extent global LV strain parameters reflect hemodynamic changes we plotted the relative change of these parameters during Dobutamine and Verapamil compared to baseline. The relative change of GLS during Dobutamine was higher than the one of CI, while the impact of Verapamil on GRS was negligible when compared to CI, overall in line with the poor reproducibility of GRS measurements. The impact of Dobutamine and Verapamil on the rest of the mechanic and hemodynamic parameters was comparable. In line with previous reproducibility studies [39–42], we showed that radial strain is a poorly reproducible and inaccurate measurement. In this study we did not show the inter- and intra-observer reproducibility because it was already assessed in a previous work from our group based on the same cohort [43]. Our analysis showed that measurements at baseline were good to excellent (good ICC 0.60–0.74; excellent ICC > 0.74) for GLS, GCS and GRS, but that only GLS and GCS displayed good reproducibility during both dobutamine and verapamil steps, whereas radial strain was highly variable [43].

### Clinical and translational perspective

In this in vivo study, we could show that CMR-FT strain parameters, such as GLS and GCS, reflect classic hemodynamic parameters such as CI, CPO, as well as a load-independent parameter of cardiac contractility such as Ees. LV strain has indeed emerged in the past year as a valid technique to assess LV deformation with high reproducibility. However, the spread of this technique in the clinical routine is limited by the often lengthy post-processing of the sequences, confining this important resource to the mere research field [44]. FT-strain, in particular, possesses the advantage of a quick assessment, being based on conventional bSSFP cine sequences [15–19], and seems to be therefore a promising technique to allow a more extensive clinical use of LV strain. Furthermore, indexing the global strain values for indirect measures of afterload, such as the invasively measured mAoP as well as the meridional wall stress, improves the ability of strain parameters to reflect LV hemodynamics. After accounting for meridional wall stress, GLS performed as good as LVEF in reflecting LV contractility, expressed as Ees, confirming the potential role of this novel parameter in the clinical arena. These results suggest that implementing strain measurements with pressure-derived variables may add accuracy to the evaluation of the mechanical and contractile function of the heart, improving the impact of LV strain in the clinical routine and helping to overcome the limitations of LVEF as a surrogate parameter of LV systolic function. In daily clinical routine, this could be potentially achieved even with a standard sphygmomanometer, as shown in the paper by Seeman at al [25].

Finally, we envision a promising role for CMR-FT LV strain investigation of chronic heart failure patients. In particular heart failure with preserved ejection fraction patients seem that they would benefit the most from this assessment, since previous studies have already shown a diagnostic and prognostic impact of strain measurements [2].

### Limitations

Specific limitation of the study are related to the fact that animals were investigated under general anaesthesia, in order to minimize the animals’ distress and to obtain stable hemodynamic conditions. Due to easier housing and milder behaviour, all the animals were females. We therefore cannot draw any relevant conclusions on the role of gender on strain variability. A limitation regarding the strain analysis is that only endocardial values of strain were analysed. Moreover, an inter-vendor software variability should be considered if looking at the absolute strain values. Another limitation is that the animals were healthy, and even if conditions of hyper-contractility and hypo-contractility were induced, these were only transient and acutely assessed. We believe the data to be representative enough for the clinical translation, however further studies are needed in a clinical setting.

In conclusion, CMR-FT derived LV strain parameters, such as GLS and GCS, correlate accordingly with LV hemodynamics in swine under various inotropic states. Indexing strain parameters for indirect measures of afterload substantially improves this correlation, with GLS being as good as LVEF in reflecting LV contractility. CMR-FT strain imaging may be a quick and promising tool to characterize LV hemodynamics in patients with a various degree of LV dysfunction.

## Data Availability

Not applicable

## Abbreviations

2Ch: Two chambers
3Ch: Three chambers
4Ch: Four chambers
BSA: Body surface area
bSSFP: Balanced steady-state free precession
CI: Cardiac index
CMR: Cardiovascular magnetic resonance
CMR-FT: Cardiovascular magnetic resonance feature tracking
CO: Cardiac output
CPO: Cardiac power output
DBP: Diastolic blood pressure
ECG: Electrocardiogram
Ees: End-systolic elastance
ESPVR: End-systolic pressure–volume relationship
FT: Feature tracking
GCS: Global circumferential strain
GCSi: Global circumferential strain indexed for mAoP
GCSw: Global circumferential strain indexed for meridional wall stress
GLS: Global longitudinal strain
GLSi: Global longitudinal strain indexed for mAoP
GLSw: Global longitudinal strain indexed for meridional wall stress
GRS: Global radial strain
GRSi: Global radial strain indexed for mAoP
GRSw: Global radial strain indexed for meridional wall stress
HR: Heart rate
LV: Left ventricle/left ventricular
LVEF: Left ventricular ejection fraction
LVESD: Left ventricular end-systolic diameter
LVP: Left ventricular pressure
mAoP: Mean aortic pressure
PWT: Posterior wall thickness
SAx: Short axis
SBP: Systolic blood pressure
SR: Strain rate
STE: Speckle tracking echocardiography
SVR: Systemic vascular resistance
TE: Echo time
TR: Repetition time
